# Determination of lead levels in maternal and umbilical cord blood at birth at the Lagos University Teaching Hospital, Lagos

**DOI:** 10.1371/journal.pone.0211535

**Published:** 2019-02-07

**Authors:** Jejelola I. Ladele, Iretiola Bamikeolu Fajolu, Veronica Chinyere Ezeaka

**Affiliations:** 1 Department of Paediatrics, Lagos University Teaching Hospital, Idi-Araba, Lagos, Nigeria; 2 Department of Paediatrics, College of Medicine, University of Lagos, Idi-Araba, Lagos, Nigeria; Holbæk Hospital, DENMARK

## Abstract

**Background:**

Lead toxicity is a cause of intellectual disability in children and majority of affected children live in developing countries. Its adverse effect on pregnancy outcome has also been documented.

**Objectives:**

To assess the relationship between maternal blood lead levels and umbilical cord blood lead levels in their corresponding newborn infants; to determine factors associated with high blood lead levels and the pregnancy outcome in participants.

**Materials and methods:**

This was a cross-sectional descriptive study carried out at a tertiary Teaching Hospital, Lagos, Nigeria. Four hundred and forty pregnant women and their respective newborns delivered at the study centre. Blood samples were obtained from the mothers and umbilical cord of the newborns at delivery and analysed by atomic absorption spectrophotometry. Socio-demographic and obstetric data was obtained by questionnaires administered to the mothers. The anthropometric measurements of the babies were taken at birth and clinical data recorded. Main outcome measures were blood lead levels in mother and baby pair, socio-demographic factors, birth weight, gestational age, length, occipito-frontal circumference.

**Results:**

The median maternal and umbilical blood lead level was 64.3μg/dl and 39.2μg/dl respectively. The levels were above 5μg/dl in 75.6% and 66.8% of mothers and umbilical cord respectively. There was a strong positive correlation between the maternal and umbilical cord blood lead levels (r_s_ = 0.80). Use of calcium supplements during pregnancy was significantly associated with a lower maternal blood lead level (*p* = 0.010) while recent painting and renovation of residential accommodation were associated with a higher umbilical cord blood lead level (*p* = 0.025). There were no statistically significant associations between the maternal and umbilical cord blood lead levels and the gestational age and anthropometry of the newborns at birth.

**Conclusions:**

The blood lead levels in newborns of women residing in Lagos, Nigeria are high and administration of antenatal calcium is associated with lower blood lead levels.

## Introduction

Lead is a recognized toxic agent in humans and the effects of its exposure and toxicity are well documented.[1–3] Lead has no known health benefit; rather it has been shown to have adverse effects on several body systems. [4,5] Children, including newborns, are particularly more vulnerable to the adverse effects of lead because of their developing brain. [2,3] Despite several attempts by many countries globally to reduce lead contamination of the environment, lead exposure and toxicity still remains a global public health problem. [2] According to the World Health Organization, lead toxicity contributes to 600,000 new cases of intellectual disabilities every year in children^6^ and 99% of children affected by lead toxicity live in the developing world[6].

Nigeria made some effort in reducing lead contamination by initiating the “phase out” of leaded gasoline in 2002 but this however, is yet to be fully achieved.[7] There is evidence of lead contamination of the soil,[8,9] air[10,11] and water[12] from previous and current leaded gasoline use, [7,13] industrialization, electronic waste,[14] refuse dumps[15] and the use of lead containing products e.g. paints,[16,17] lead acid batteries[18] and eye cosmetics (“*tiro/kohl”)*.[19,20] Studies done on some Nigerian foods such as vegetables,[21] roasted meat, smoked fish,[22,23] dried meat, and canned beverages[24] have also reported high lead levels. In Lagos State, over the last few years, the widespread use of generators powered by leaded gasoline has become an epidemic due to erratic power supply. Furthermore, there has also been a massive influx of a variety of transportation options to match the increasing population growth, which include motorcycles and tricycles both of which are also powered by gasoline[25].

Pregnant women and their unborn fetuses are vulnerable groups exposed to a higher lead concentration because of the physiologic mobilization of bone lead stores during pregnancy and lactation.[26] Prenatal lead exposure can occur as lead moves readily from mother to fetus via passive diffusion. [2,3,26,27] Lead has been detected in the fetal brain as early as the 13^th^ week of gestation[28] and the effects on pregnancy outcome are seen across a wide range of maternal blood lead levels.[27] These effects include gestational hypertension,[29,30] spontaneous abortion,[31] preterm delivery,[32] congenital anomalies,[27] low birth weight,[33,34] decreased length and head circumference.[35] The most critical consequence of prenatal and childhood lead exposure is poor neuro-developmental outcome especially in cognitive functioning even at low doses irrespective of lead exposure during other life stage. [2,36–41] Other effects are shortening of attention span and disruption of behavior.[41–43] There appears to be no threshold level below which lead causes no injury to the developing human brain[26,27].

There are currently no internationally recognized guidelines on the prevention and management of lead poisoning worldwide. Thus, countries set their own guidelines for levels of lead exposure that they consider as safe.[44] In developed countries, routine testing of blood lead levels (BLL) is recommended in pregnant women and/or at-risk communities.[26] Risk factors for lead exposure identified in pregnant women include pica, recent immigration, occupation, nutritional status, use of alternative remedies and cosmetics and use of lead glazed pottery for cooking and storing food.[26] In the United States, the Centers for Disease Control and Prevention (CDC) recommends follow up testing for both pregnant women and their newborn infants if BLL is ≥5μg/dl, and chelation therapy is instituted on a case to case basis if BLL > 45μg/dl[26].

Although there have been numerous studies on prenatal lead exposure and pregnancy outcomes worldwide,[2,3,26,45] there is paucity of data on prenatal lead exposure and its effects on children in Nigeria. Nigeria recorded the world’s worst case of lead poisoning in 2010 in Zamfara State where an estimated 400 children died from lead toxicity following exposures from mining sites.[46] In May, 2015 there was an outbreak of lead poisoning in Niger State which killed 28 children of the 65 confirmed cases.[47] The prevalence of prenatal lead exposure using maternal blood in Nigeria has been reported as ranging from 78.9% to 100% [48–50] and that in children aged six months to nine years ranged from 25% to 97%.[46,51–54] It is evident that environmental lead contamination remains a major challenge in Nigeria and there is an urgent need to initiate policy on environmental lead contamination and establish screening and management guidelines.

Hence, this study aimed to determine the lead levels in maternal and umbilical cord blood at birth.

## Materials and methods

### Study location

The study was conducted at the Labour ward complex and Neonatal unit of the Lagos University Teaching Hospital (LUTH), Idi-Araba, Lagos, Nigeria. Lagos is highly urbanized and densely populated, and it is one of the fastest growing cities in the world. Being a former national capital, it remains the nation’s leading economic and commercial hub as it houses 60% of the country’s manufacturing companies hence represent fertile grounds for environmental lead exposure in the population. The Lagos University Teaching Hospital is a 760 bed Federal tertiary hospital providing healthcare to inhabitants of Lagos state and most neighbouring states. The hospital has two obstetric wards, a labour ward complex comprising of 12 delivery suites and two operating suites.

### Sample size determination

Concerning the subject recruitment; the sample size was derived using the formula described by Kirkwood for studies involving single mean.

$$\text{n}={\text{SD}}^{\text{2}}/{\text{SE}}^{\text{2}}$$

Where; n = minimum sample size, SD = standard deviation and SE = standard error.

This study involved the measurement of lead in both maternal and umbilical cord blood. Attention was focused on each parameter separately. Since there were no local published studies on lead in umbilical cord blood, the outcome from a local maternal lead blood level was used. A standard deviation of 1.0 as determined by Njoku and Orisakwe[48] who studied lead levels in pregnant women in an urban setting in Nigeria in 2012 was used to determine the sample size. Where; SD = standard deviation = 1, SE = standard error = 0.05:

$${\left(1.0\right)}^{2}/{\left(0.05\right)}^{2}=400$$

The highest sample size calculated was 400. This was increased by 10% in order to account for loss of samples during processing and/or possible withdrawal of consent. Hence, an additional 40 subjects were added making a total of 440 mother- umbilical cord pairs recruited for the study.

### Institutional ethical approval

The study protocol was approved by the Health Research Ethics Committee of the Lagos University Teaching Hospital before commencement of the study. Written informed consent was also obtained from all participants.

### Subject recruitment

Detailed information about the study was given to all women who presented to the labour ward and labour ward theatres for delivery and those that gave informed written consent were consecutively recruited until sample size was reached. Thereafter, the pre-tested study questionnaire was administered to them to obtain their bio-data, socio-demographic and obstetric history. Those that were brought in with emergent conditions such as ecclampsia, uterine rupture, ante-partum haemorrhage, pregnant women with sickle cell anaemia, those on HRT or mega doses of vitamin C and those who did not consent were excluded.

### Subject handling procedure

Following delivery, the baby’s anthropometry i.e. birth weight, length and occipito-frontal circumference were measured. The birth weight was obtained using a bassinet weighing scale by Weighmaster D.127 serial number 64555. Reliability of results was guaranteed by ensuring weekly standardization of the scale using known weights. Length was measured using an infantometer by Olympic Surgical Company Inc., Washington 98101. Baby was placed in a supine position on the infantometer; the head was held in place and an assistant ensured that the knees were extended before the lengths were then read in centimeters to one decimal. Occipito-frontal circumference was measured using an inelastic tape measure placed around the head with the supraorbital ridge and occipital prominence as the landmarks. The gestational age was determined using the last menstrual period (LMP) which was corroborated with the Ballard score; in cases of discrepancy baby was excluded from the study. Appropriateness of the baby’s weight for gestational age was determined using the standards of intrauterine growth for an African population at sea level described by Olowe[55].

### Blood sample collection

Two millilitres of maternal venous blood was drawn prior to delivery and umbilical cord blood was taken at delivery prior to cutting of the cord. Both samples were collected using lead free needle and syringes and immediately transferred into metal free potassium EDTA vacutainer tubes (medi-scan technology 10102, Florida USA) to prevent coagulation. The samples were preserved in the unit refrigerator at 4°c before transportation to the Central Research Laboratory, University of Lagos within 24hours where they were processed and stored in the freezer at -80°C prior to blood lead analyses.

### Blood lead analysis

Total blood lead level was detected using the Perkin Elmer Analyst 400 Atomic Absorption Spectrometer (Waltham, USA) with air-acetylene gas mixtures via a hollow cathode lamp at spectrum of 283.3nm.[56] Before analysis, the blood was digested with 10ml concentrated HN0_3_. In order to minimize errors and ensure reliability of results, all glassware used in analyses were cleaned and washed with HNO_3_ prior to use and decontaminated after each use for the next batch. Also, prior to blood lead analyses, the machine was first auto-zeroed using the blank sample. Thereafter four standards of known concentrations (5mg/L, 10mg/L, 20mg/L, and 40mg/L) were run against the blank to generate data to determine the calibration curve. The blanks were run often to verify calibration in the course of the entire analyses. Once the calibration was done, lead measurements in each blood sample were run twice and the mean of the two results was recorded as the final result of that sample.

### Statistical analysis

Data was analysed using the Statistical Package for the Social Sciences (SPSS) for Windows version 17. Descriptive statistics were reported as mean ± SD in un-skewed data while skewed data was reported as median (range). Categorical data was expressed as frequencies and percentage distribution. Socio-demographic and obstetric comparisons with varying concentration of lead levels in the maternal and cord blood was done using Student t-test and chi-square test (as appropriate for continuous and categorical data respectively). Fisher exact test was used to analyse data when categories had expected numbers less than five. Spearman’s correlation coefficient (r_s_) was used to demonstrate correlation between the maternal blood lead levels and that of the umbilical cord, and maternal and umbilical cord blood levels and the newborns anthropometry. The level of statistical significance was set at p <0.05.

## Results

Four hundred of 440 mother-newborn pairs recruited for the study had complete maternal-cord blood samples and questionnaires and were included in the analysis. Table 1 shows a summary of the socio-demographic characteristics of the mothers.

**Table 1:** Maternal socio-demographic characteristics.

Variable	n (%) (N = 400)
**Maternal Age (years)**	
**<25**	54 (13.5)
**25–29**	116 (29.0)
**30–34**	137 (34.2)
**>35**	93 (23.3)
**Mean ± SD(years)**	30.3 ± 5.2
**Educational level**	
**None**	3 (0.8)
**Primary**	28 (7.0)
**Secondary**	140 (35.0)
**Tertiary**	229 (57.2)
**Maternal occupational exposure**	
**No**	391 (97.8)
**Yes**	9 (2.2)
**Ethnicity**	
**Yoruba**	244 (61.0)
**Ibo**	112 (28.0)
**Hausa**	5 (1.2)
**Others**	39 (9.8)
**No. of Years in present Residence**	
**<1year**	101 (25.2)
**≥1year**	299 (74.8)
**Painting of house**	
**No**	251 (62.8)
**Yes**	149 (37.2)
**Location of residence**	
**Badagry**	22 (5.5)
**Epe**	48 (12.0)
**Ikeja**	96 (24.0)
**Ikorodu**	5 (1.2)
**Lagos**	229 (57.3)
**Residence on a major road**	
**No**	252 (63.0)
**Yes**	148 (37.0)
**Source of drinking water**	
**Borehole**	54 (13.5)
**Bottle water**	194 (48.5)
**Public supply**	23 (5.7)
**Sachet water**	128 (32.0)
**Well**	1 (0.3)

Figures in brackets are in percentages

### Obstetric characteristics and risk factors for lead exposure

Majority of the mothers were multiparous (74.8%). Significantly higher proportion (82.2%) of women attended antenatal care during pregnancy and 38.5% took calcium supplements regularly during pregnancy. Almost six percent of the pregnant women recruited practiced pica during pregnancy and most of those who practiced pica ate “*nzu”* also known as native chalk. A significant proportion (29.0%) of the respondents took herbal mixtures during pregnancy and 7.8% took alcohol. One percent of those recruited were active smokers while 8.2% were passive smokers. Hypertension in this study was made up of gestational hypertension and pre-eclampsia. Mothers who had chronic hypertension were not captured. Sixty (15%) of the women developed hypertension during pregnancy.

The association between some maternal obstetrics and socio-demographic characteristics on pregnancy outcome is shown in Table 2. Maternal gravidity greater than 1 and non-attendance of ANC were the only factors significantly associated with OFC less than the 3^rd^ centile.

**Table 2:** Maternal obstetrics and socio-demographic characteristics and pregnancy outcome.

Variables	Preterm birth	Birth weight <3^rd^centile	Birth length <3rd centile	Birth OFC <3rd centile
**Maternal age**				
<35yrs (307)	45 (14.7%)	13 (4.2%)	31 (10.1%)	41 (13.4%)
≥35yrs (93)	13 (13.9%)	8 (8.6%)	8 (8.6%)	11 (11.8%)
p-value	0.233	0.098	0.67	0.701
**Maternal education**				
No formal /1° (31)	4 (12.9%)	18 (4.9%)	35 (9.5%)	46 (12.5%)
2°/3° (369)	54 (14.6%)	3 (9.7%)	4 (12.9%)	6 (12.4%)
p-value	1.000	0.216	0.526^+^	0.268^+^
**Gravidity**				
1 (101)	11 (10.9%)	5 (5.0%)	11 (10.9%)	20 (19.8%)
>1 (299)	47 (15.7%)	16 (5.4%)	28 (9.4%)	32 (10.7%)
p-value	0.233	0.876	0.655	0.019*
**Gestational HT**				
Yes (60)	10 (16.7%)	2 (3.3%)	8 (13.3%)	9 (15.0%)
No (340)	48 (14.1%)	19 (5.6%)	31 (9.1%)	43 (12.6%)
p-value	0.605	0.753 ^+^	0.31	0.617
**ANC** (329)	46 (14.0%)	14 (4.3%)	28(8.5%)	34 (10.3%)
No ANC (71)	12 (16.9%)	7 (9.9%)	11 (15.5%)	18(25.4%)
p-value	0.526	0.074^+^	0.072	0.002*
**Cigarette smoke**				
Exposure (37)	9 (25.7%)	3 (8.6%)	4(11.4%)	4(11.4%)
No exposure (363)	49 (13.4%)	18 (4.9%)	35(9.6%)	48(13.2%)
p-value	0.074	0.414^+^	0.764^+^	1.000^+^
**Alcohol intake**				
Yes (31)	8 (13.6%)	0 (0%)	4(112.9%)	4 (13.0%)
No (369)	50(25.8%)	21 (5.7%)	35(9.5%)	48 (12.9%)
p-value	0.105^+^	0.392^+^	0.526^+^	1.000^+^

*Significant p-value;

^+^ Fisher’s exact test

General characteristics of the newborns are depicted in Table 3. A total of 192 males and 208 females were studied. Birth weight ranged from 800g to 5250g. Eighty three percent of the babies were term while 48 (12.0%) had low birth weights. Among the pre-terms, two babies had extreme low birth weights (below 1000 grams). The mean gestational age, birth weight, length and occipito-frontal circumference at birth were 38.2±2.5, 3104±679.4, 48.1±3.7 and 34.2±2.3 respectively.

**Table 3:** Gestational age and anthropometry of study participants.

Variables	N = 400 n (%)
**Gestational age (weeks)**	
**<37weeks**	58 (14.5)
**37-41weeks**	335 (83.8)
**>41weeks**	7.(1.7)
**Mean ± SD (weeks)**	38.2±2.5
**Birth weight (grams)**	
**<1000**	2 (0.5)
**1000–1499**	9 (2.2)
**1500–2499**	37 (9.3)
**2500–4000**	326 (81.5)
**>4000**	26 (6.5)
**Mean ± SD (grams)**	3104.7±679.4
**Birth length (centimetres)**	
**<3**^**rd**^ **centile**	40 (10.0)
**3**^**rd**^ **-97**^**th**^ **centile**	336 (84.0)
**>97**^**th**^ **centile**	24 (6.0)
**Mean ± SD (centimetres)**	48.1±3.7
**OFC (centimetres)**	
**<3**^**rd**^ **centile**	52 (13%)
**3**^**rd**^- **97**^**th**^ **centile**	344 (86%)
**>97**^**th**^ **centile**	4 (1%)
**Mean ± SD(centimetres)**	34.2±2.3

**Fig 1:**
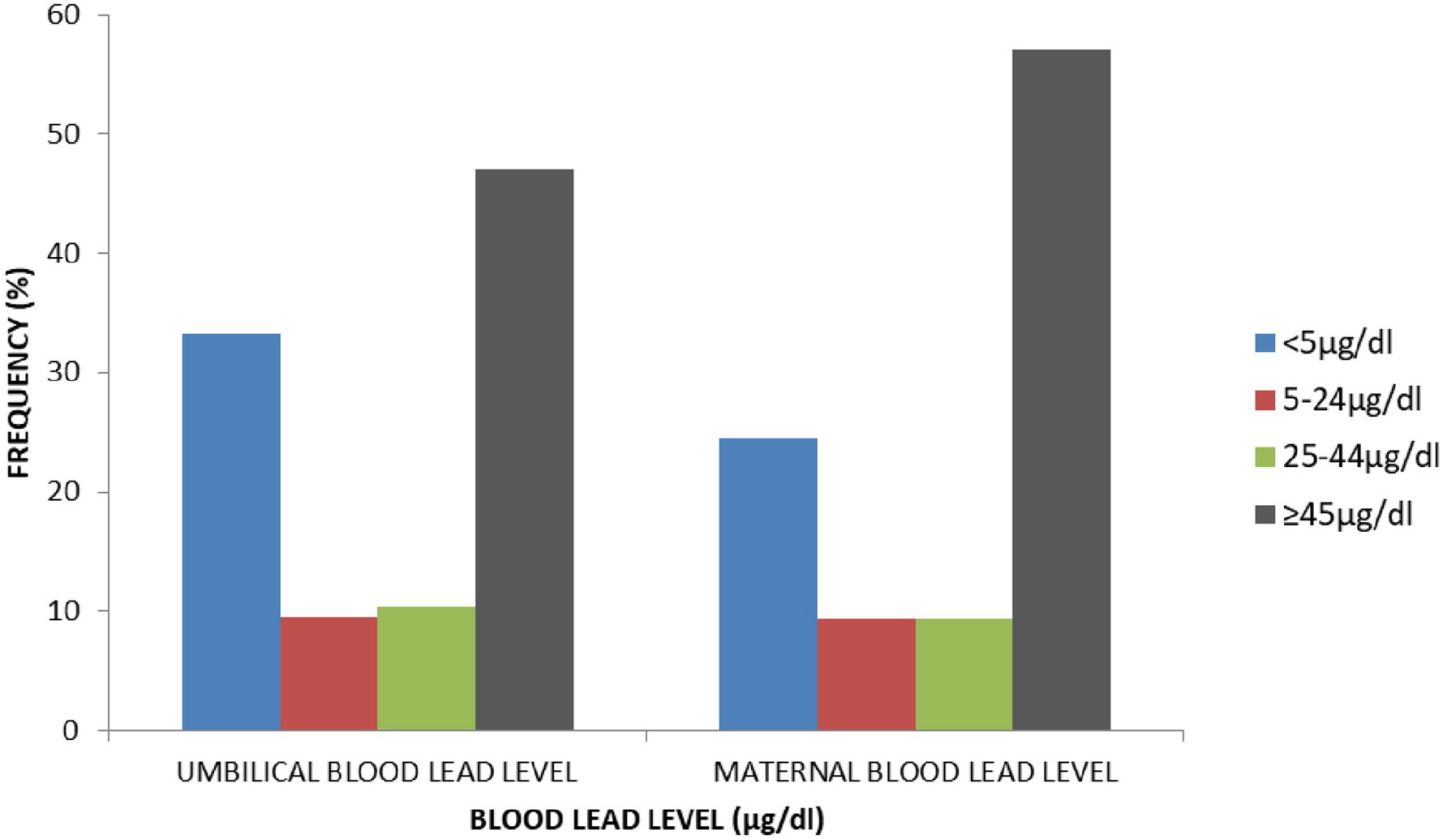
Distribution of lead levels in maternal and umbilical cord blood.

Fig 1 shows the distribution of lead levels in maternal and umbilical cord blood based on levels for intervention and the percentage prevalence of blood lead levels. Elevated levels were observed in 75.6% of maternal and 66.8% of umbilical cord blood samples. Thirty three percent of cord samples had values less than 5μg/dl. Over half (57.0%) of mothers had lead levels in the range of pharmacological intervention and hence could be termed as high risk pregnancies.

**Fig 2:**
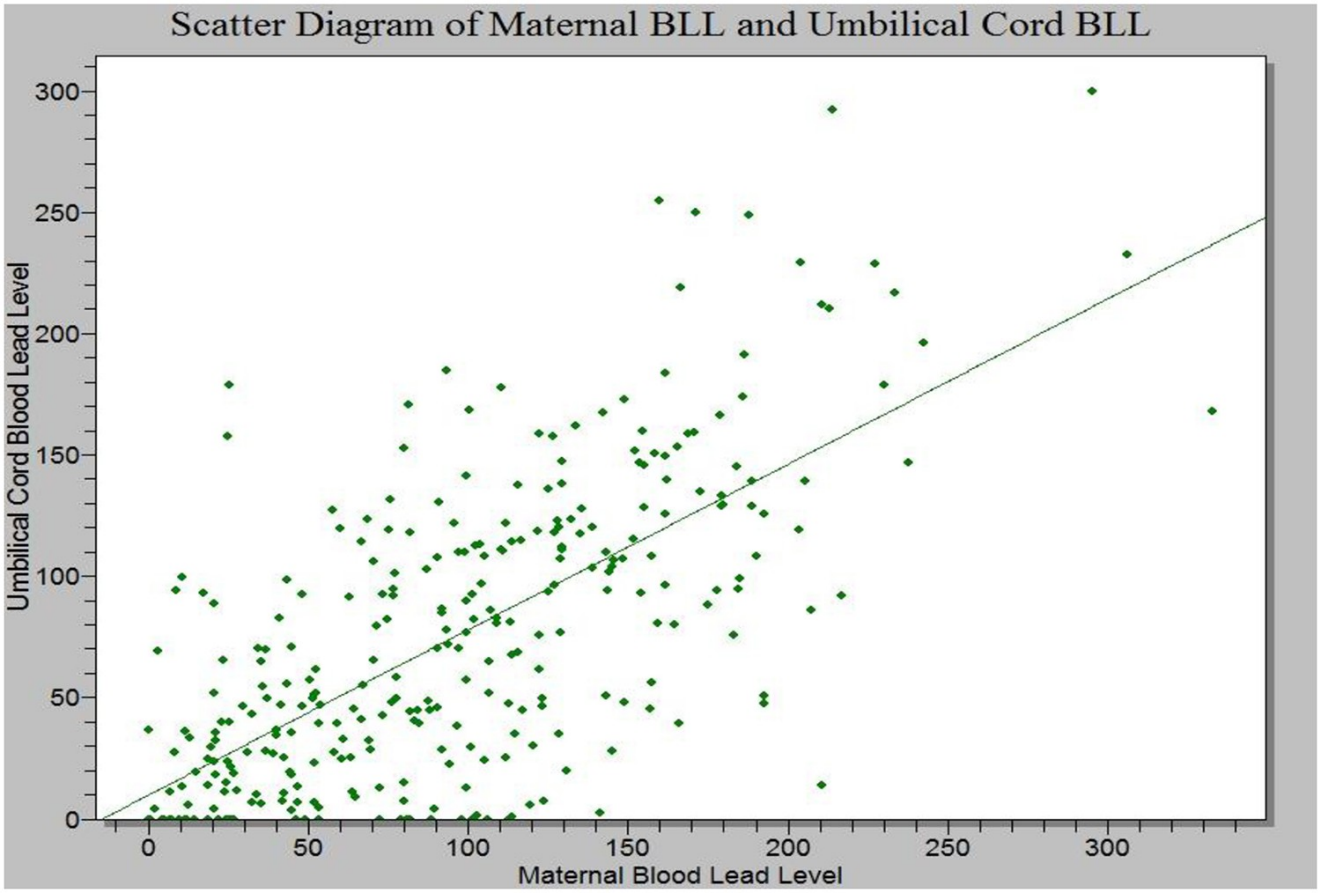
Correlation between maternal and umbilical cord blood lead levels.

The relationship between the maternal and umbilical cord blood lead levels is illustrated in Fig 2. This scatter diagram shows a positive correlation between the two variables, with a significant coefficient of correlation (r_s_ = 0.80, F = 587.8 *p* = 0.000).

Table 4 shows the relationship between pregnancy outcome and maternal and umbilical cord blood lead levels. There was no significant difference in pregnancy outcomes when outcome for high umbilical blood and maternal blood lead levels were compared with normal levels.

**Table 4:** Association between pregnancy outcome and maternal and umbilical cord blood lead levels.

Pregnancy outcome	Umbilical blood lead levels		p-value	Maternal blood lead levels		p-value
	Positive	Negative		Positive	Negative	
	N (267)	N (133)		N (267)	N (133)	
**Gestation**			0.932			0.555
**Preterm**	39	19		42	16	
**Term**	228	114		260	82	
**Birthweight**			0.151			0.334
**<3rd centile**	11	10		14	7	
**>3rd centile**	256	123		288	91	
**Length**			0.712			0.338
**<3rd centile**	25	14		27	12	
**> 3rd centile**	242	119		275	86	
**OFC**			0.137			0.141
**< 3rd centile**	30	22		35	17	
**> 3rd centile**	237	111		267	81	

Positive = lead values ≥ 5ug/dl, Negative = lead values < 5ug/dl

Table 5 illustrates the association between maternal lead levels and socio-demographic factors; lower maternal age, Ibo tribe and recent painting and renovation were associated with higher maternal blood lead levels. However, following analysis these did not reach statistical significance. Other correlates such as level of education, number of years lived in present home and living in close proximity to a major road did not have any statistically significant effect on the concentration of lead in the mothers. Table 6 shows bivariate analysis of risk factors for prenatal exposure and maternal blood lead levels. The only statistical significant variable was the use of calcium supplements during pregnancy. Mothers who took calcium had lower lead values compared to those that did not. (*p* = 0.01). Multiparous women were seen to have higher lead levels compared to those of lower gravid status but this finding was not statistically significant.

**Table 5:** Relationship between maternal blood lead levels and the socio-demographic factors.

Variables	Maternal Lead Levels		Statistics	*p* value
	Positive n = 302 (%)	Negative n = 98 (%)		
**Mother’s age (N)**				
**<25 (54)**	42 (77.8)	12 (22.2)	χ^2^ **=** 1.4	0.711
**25–29 (116)**	89 (76.7)	27 (23.3)		
**30–34 (137)**	105(76.6)	32 (23.4)		
**>35 (93)**	66 (71.0)	27 (29.0)		
**Mean Age ± SD**	30.2±5.2	30.9±5.2	t = 1.2^#^	0.225
**Educational level**				
**None (3)**	2 (66.7)	1 (33.3)	χ^2^ **=** 0.6	0.806^+^
**Primary (28)**	20 (71.4)	8 (28.6)		
**Secondary (140)**	108(77.1)	32 (22.9)		
**Tertiary (229)**	172(75.1)	57 (24.9)		
**Positive maternal occupational exposure (9)**	7 (77.8)	2 (22.2)	χ^2^ **=** 0.0	1.000^+^
**Ethnicity**				
**Yoruba (244)**	180(73.8)	64 (26.2)	χ^2^ = 4.6	0.151^+^
**Ibo (112)**	92 (82.1)	20 (17.9)		
**Hausa (5)**	3 (60.0)	2 (40.0)		
**Others (39)**	27 (69.2)	12 (30.8)		
**>1year in present residence (299)**	224(74.9)	75 (25.1)	χ^2^ = 0.2	0.373
**Recent painting of house (154)**	124(80.5)	30 (19.5)	χ^2^ = 2.8	0.059
**Location of Residence**				
**Badagry**	18 (82.0)	4 (18.0)	χ^2^ = 2.1	0.872^+^
**Epe**	34 (70.8)	14 (29.2)		
**Ikeja**	70 (72.7)	26 (27.1)		
**Ikorodu**	4 (80.0)	1 (23.1)		
**Lagos**	176 (76.9)	53 (23.1)		
**Residence on a major road (148)**	108 (73.0)	40 (27.0)	χ^2^ = 0.8	0.217
**Source of drinking water**				
**Borehole**	40 (74.1)	14 (25.9)	χ^2^ = 2.4	0.685^+^
**bottle water**	143 (73.7)	51 (26.3)		
**public supply**	20 (87.0)	3 (13.0)		
**sachet water**	98 (76.6)	30 (23.4)		
**Well**	1 (100.0)	0.(0.0)		

Positive = lead values ≥ 5ug/dl, Negative = lead values < 5ug/dl,

^#^student t test,

^+^Fishers’ exact *p* value

**Table 6:** Association between risk factors for lead exposure and maternal blood lead levels.

Variables	Maternal blood lead levels		χ2	p-value
	Positive (302)	Negative (98)		
	n(%)	n(%)		
Gravidity			3.2	0.204
1	2(81.2)	19 (18.8)		
2	76 (76.8)	23 (23.2)		
3	144 (72.0)	56 (28.0)		
Use of calcium supplement	106 (68.8)	48(31.2)	6	0.010*
Positive pica practice	18 (78.3)	5 (21.7)	0.1	0.489
Use of clay pot			0.4	1.000
Yes	296 (75.3)	97 (24.7)		
No	6 (85.7)	1 (1)		
Active Smoking	3 (75.0)	1.(25.0)	0	1.000+
Passive Smoking	25 (75.8)	8 (24.2)	0	0.581
Alcohol intake	22 (71.0)	9 (29.0)	0.4	0.338
Herbal intake	84 (72.4)	32 (27.6)	0.8	0.214
Hypertension in pregnancy	45 (75.0)	15 (25.0)	0	0.518
Use of tiro ^a^	59 (72.0)	23 (28.0)	0.7	0.242
Canned food intake	176(73.0)	65 (27.0	2	0.097

Positive = lead values ≥ 5ug/dl, Negative = lead values < 5ug/dl,

^+^Fishers’ exact *p* value.

*p value statistically significant,

^a^ eye cosmetic similar to Kohl

Depicted in Table 7 below is the association between the various socio-demographic factors and umbilical cord blood lead levels. The proportion of UCBLL was similar across all maternal levels of education. Nine mothers were working directly in a lead related occupation. Of these nine, six (66.7%) had elevated umbilical cord blood levels. However, this was not statistically significant (*p* = 0.62) as mothers who were not directly involved in a lead related job equally had elevated UCBLL. One hundred and fourteen fathers worked in lead related occupations. Cord blood samples from the wives of these men showed that 84 (73.7%) had lead levels above 5μ/dl and 30 (26.3%) had levels below 5μg/dl. This finding however was not statistically significant (*p* = 0.05).

**Table 7:** Maternal and paternal socio-demographic variables and the umbilical cord lead levels.

Variable	Umbilical cord blood level		χ2	p-value
	Positive (267))	Negative (133)		
	n (%)	n (%)		
Mother’s age			2.4	0.487
<25	40 (74.1)	14 (25.9)		
25–29	74 (63.8)	42 (36.2)		
30–34	94 (68.6)	43 (31.4)		
>35	59 (63.4)	34 (36.6)		
Education			0.7	0.861+
None	2 (66.7)	1 (33.3)		
Primary	17 (60.7)	11 (39.3)		
Secondary	96 (68.6)	44 (31.4)		
Tertiary	152 (66.4)	77 (33.6)		
Maternal occupational exposure	6 (66.8)	3 (33.3)	0	1.000+
Ethnicity			5.9	0.089+
Yoruba	155(63.5)	89 (36.5)		
Ibo	85 (75.9)	27 (24.1)		
Hausa	3 (60.0)	2 (40.0)		
Others	24 (61.5)	15 (38.5)		
Paternal occupational exposure	84 (73.7)	30 (26.3)	2.8	0.057
>1year in present Residence	195 (65.2)	106 (34.8)	1.3	0.159
Recent painting of house	110 (73.7)	39 (26.3)	4.2	0.025*
Source of water			4.4	0.347+
Borehole	33 (61.1)	21 (38.9)		
Bottle water	126 (64.9)	68 (35.1)		
Public supply	19 (82.6)	4 (17.4)		
Sachet water	88 (68.8)	40 (31.3)		
Well	1 (100.0)	0 (0.0)		

Positive = lead values ≥ 5ug/dl, Negative = lead values < 5ug/dl,

^+^Fishers’ exact p value

*p value statistically significant

As regards ethnicity, the Ibos recorded the greatest proportion of elevated cord lead levels 75.9%. There was no significant difference in the number of years spent in the subject’s place of residence (*p* = 0.15) and the umbilical cord blood lead level. Nonetheless this study recorded a higher proportion of elevated cord lead samples in women who had lived in their present place of abode for less than a year. Recent painting / renovation within the last one year was statistically significant (*p* = 0.025).

For ease of analysis, the area of residence was divided into five main administrative areas. Majority of the subjects reside in Lagos and Ikeja divisions. The higher proportions of elevated lead levels were however found in those residing in Badagry and Ikorodu though this finding was of no statistical significance (*p* = 0.57). The study centre is located within the Lagos division and explains why most of the subjects recruited attended the centre for their ante-natal/delivery. The proximity of residence to a major road was not significantly associated with the umbilical cord blood lead levels (*p* = 0.17). Also no significant association was observed between sources of drinking water and the cord lead levels (*p* = 0.35). This study however shows that 100% of women who drank well water and 82.6% of those that drank public water had higher umbilical cord values as opposed to those that took bottled water and sachet water.

There were no significant associations seen between the mother’s obstetric factors and the presence of high cord blood lead levels as shown in Table 8 below. Higher cord blood lead levels were seen in first time mothers than multiparous mothers; however, this was not statistically significant. *(p* = 0.53). The study also found that antenatal care attendance did not influence the cord blood lead levels neither did the use of calcium during pregnancy. It is worthy to note that 62.5% of pregnant women who did not use calcium during pregnancy had higher cord lead levels as opposed to 37.5% who took it. Pica, smoking either passively or actively and hypertension were associated with higher cord blood lead levels but all these did not reach the level of significance. Alcohol intake, *tiro* and herbal use had no effect on the concentration of lead in the umbilical cord.

**Table 8:** Associations between maternal obstetric and risk factors for lead exposure and umbilical cord blood levels.

Variables	Umbilical cord blood lead levels		χ2	p-value
	Positive (267)	Negative (133)		
	n (%)	n (%)		
Gravidity				0.531
1 (101)	72 (71.3)	29 (28.7)	1.3	
2 (99)	65 (65.7)	34 (34.3)		
3 (200)	130 (65.0)	70 (35.0)		
No Antenatal care (71)	44 (62.0)	27 (38.0)	1.4	0.507
No Antenatal calcium intake (246)	173(70.5)	73 (29.5)	2.9	0.055
Positive pica practice (23)	17 (73.9)	6 (26.1)	0.6	0.307
Use of clay pot (7)	6 (85.7)	1 (14.3)	1.2	1.000^+^
Smokes actively (4)	3 (75.0)	1 (25.0)	0.1	1.000^+^
Smokes passively (33)	24 (72.7)	9 (27.3)	0..6	0.29
Takes alcohol (31)	20 (64.5)	11 (35.5)	0.1	0.426
Positive pica practice (23)	17 (73.9)	6 (26.1)	0.6	0.307
Positive use of herbs(116)	73 (62.9)	43 (37.1)	1.1	0.179
Hypertension in pregnancy(60)	41 (68.3)	19 (31.7)	0.1)	0.451
Use of Tiro (82)	49 (59.8)	33 (40.2)	2.3	0.085
Intake of canned food (241)	149(61.8)	92 (38.2	6.6	0.007^+^*

Positive = lead values ≥ 5ug/dl, Negative = lead values < 5ug/dl,

^+^Fishers’ exact *p* value,

*p value statistically significant

**Fig 3:**
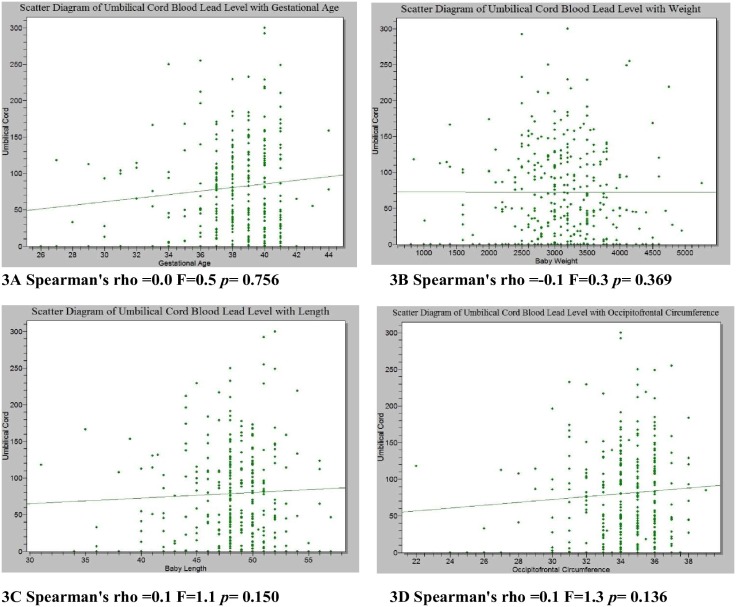
(A-D).Correlation between umbilical cord blood lead level and gestational age and anthropometric values.

Linear regression analysis using Spearman’s correlation was carried out to explore possible associations between umbilical cord blood lead levels and the gestational age and anthropometry of the newborns. There was no correlation between the lead level and these variables as depicted in Fig 3A–3D.

## Discussion

The median blood lead level obtained for the mothers was 63.7μg/dl (range of 0–332μg/dl) and this is similar to the value reported in another study in Lagos.[57] This value is 12 times higher than the current intervention cut off value of 5μg/dl. This finding may likely be due to the higher levels of lead contamination in Lagos environment as published by several authors[7,21,58,59,60,61] which invariably translates to a high exposure level to the inhabitants. Several factors such as emissions from vehicles, tricycles, motorcycles, generating sets and industrial emissions, widespread construction and use of lead based paints account for the higher lead contamination. The possible implication of this is that there is a high risk of severe health effects of lead on pregnant women and their off-springs in Lagos.

The median lead level in umbilical cord blood obtained in the current study is high and this is a reflection of maternal lead concentration. This high level is in contrast to reports from developed countries where umbilical cord blood lead levels have gradually declined over the years following the phasing out of leaded gasoline and the ban of lead paints and lead containing products with continuous bio monitoring.[62,63] These countries have implemented several strategies aimed at reducing environmental lead.

The prevalence rates of elevated lead levels in maternal and umbilical cord blood in this study were high, 75.6% and 66.8% respectively. This is similar to the values obtained by Njoku and Orisakwe[48] who reported a prevalence rate of 78.9% in pregnant women in Imo state but lower than that reported in Abakaliki[49] where a higher prevalence rate of 88.5% was documented. The lower prevalence seen in the current study compared to the Abakaliki study is unexpected. One would have anticipated a higher rate considering the study location which has a higher ambient lead contamination and the less selective nature of the study population compared to other Nigerian studies. Certain practices like ante-natal calcium supplementation which has been reported to be protective against lead toxicity may have contributed to the lower prevalence. Forty percent of study participants were on calcium supplements. Although mothers in the other studies were also attending ante-natal clinics, their status as regards calcium use was not stated.

In the current study, there was a strong positive correlation between maternal and umbilical cord blood lead level (r_s_ = 0.80, *p* = 0.000) which infers that maternal blood lead level may be a sufficient marker of prenatal exposure. This result is in tandem with the report of other researchers which showed that there was a direct relationship between maternal and umbilical cord blood lead levels.[64,65,66] Higher lead levels were found in maternal samples compared to umbilical cord samples as seen in most studies. However, 18% of the umbilical cord samples had higher lead values than their maternal counterparts similar to the prevalence reported by other authors.[62,67] The current study found that four percent of those with a higher umbilical cord blood lead level with respect to maternal cord blood lead level had an elevated blood pressure and none of them were active smokers. More studies need to be carried out to explore factors that may be responsible for this effect.

In about a quarter of the study population, fathers were occupationally exposed to lead. The umbilical cord blood lead levels were higher in participants whose fathers were occupationally exposed to lead compared to those not exposed. High UCBLL may probably be due to high maternal blood lead levels following second hand exposure through the fathers. A similar result which showed a positive association between the husband’s occupation in the lead industry and elevated maternal lead level independent of place of residence has been previously reported.[68] An expanded study of the current population to include fathers lead level may show a similar trend.

The mother’s level of education was not shown to have any association with maternal and umbilical cord blood lead concentration in this study. This is comparable to what some previous investigators have reported[62] in which umbilical cord blood lead had no association with the level of education. Some other researchers had a contrasting result showing an inverse relationship between the level of education and lead level i.e. higher concentration noted in women with a lower level of education. The level of education has been used as an indirect measure of socio-economic class with higher lead levels seen in women from poorer backgrounds according to studies by other authors.[1,69] A suggested reason for this difference is probably because the current study centre is a tertiary fee paying hospital thus fewer women with lower educational status present here for their health care needs.

The use of calcium supplements by the mothers during pregnancy was of significant importance; with lower maternal blood lead concentration being associated with calcium supplementation in pregnancy. This also had an effect on umbilical cord blood lead levels with figures approaching statistical significance. Calcium requirements are known to increase during pregnancy (especially in the third trimester) and lactation due to the high demand by the fetus and the effect of calcium enriched diet and calcium supplementation on lead availability has been studied extensively in animals and humans. While some studies including that carried out on Nigerian children have found no association, majority however have measured decreases in the blood lead concentration of calcium supplemented women.[70,71] Both sets of authors[71,72] concluded that calcium supplementation may be beneficial in reducing BLL in pregnant women with elevated blood lead. Ante-natal calcium supplementation recommended by the WHO for prevention of pre-ecclampsia in populations where calcium intake is low is currently practiced in LUTH. This intervention may play an additional beneficial role in reducing lead toxicity by preventing the mobilization of lead from maternal bone.

Recent household painting and renovation/remodeling were significantly associated with elevated UCBLL in this study. Lead in paint has been described as one of the most common sources of exposure in children however adults including pregnant women can be exposed to paint dust from house renovation and remodeling of homes.[2] During renovation, lead dust can be released into the air and persists in that environment. Lead paint hazard includes not only lead paints but also lead found in settled dust and bare soil. Studies[16,58] on household paints in Nigeria have reported elevated lead content with prevalence rates ranging from 83%-96% indicating that almost every home in Nigeria has the potential to cause lead exposure and even lead toxicity. The result in this study was therefore, not surprising. There is currently no regulation on lead containing paints in Nigeria, the Standard Organization of Nigeria (SON) in 2015.set the standard of a maximum of 90ppm for lead in paints, but this is yet to receive council approval and be gazetted and fully enforced[73].

This current study demonstrated higher maternal and umbilical cord blood lead levels in participants residing in Badagry and Ikorodu areas of Lagos and although these subjects accounted for only 6.6% of the study population, the highest maternal and cord blood levels were recorded in these groups of participants. The possibility of high environmental pollution in these areas warrants environmental research and monitoring to investigate possible source(s) and institute remediation if necessary.

Source of drinking water did not show any association with both maternal and cord blood levels similar to findings by other researchers.[69] In the current study, the major sources of water were bottled and sachet water. Studies carried out on ground water and sachet water in Lagos by previous investigators[12,60,72].all showed lead levels ranging between 0.05–0.24mg/L which is above the permissible limit of 0.01mg/L for water. This lack of association in this study may be attributed to exposure misclassification as responders may not be aware of the origin of their water source. The actual sources of bottled and sachet water are variable and may be unknown to the consumers.

No association was found between maternal and cord blood lead levels and residence in close proximity to a major road. Most studies that reported an association were carried out in the era of leaded gasoline use and hence it was deduced that vehicular emissions from heavy traffic was responsible for this effect. As most of the participants in this study were employed in either the public or private sector, they were likely to spend most of their day time out of their homes. This could have contributed to the lack of association.

High maternal blood lead was noted in multiparous women and in those that smoked cigarettes. The effects of smoking in pregnancy and lead concentrations in both the mothers and fetuses have been studied by a number of researchers.[74,75] Smoking is a risk factor for lead toxicity as lead is found in some cigarettes and also cigarette smoking is associated with certain micronutrient deficiencies. Smoking in women especially during pregnancy is uncommon in this part of the world; hence it is not unexpected that only a small percentage of the women studied were active smokers. Majority of documented exposure to cigarettes was through passive smoking thus this could explain why a significant association was not found in the current study. Unexpectedly, there was a significant association between umbilical cord lead and the intake of canned food. Lead solder used in cans is thought to contribute to the lead burden when ingested.^2,24^ Other factors such as antenatal care attendance, alcohol consumption, herbal intake and use of lead containing eye cosmetic “*tiro”* were not associated with lead levels in this study.

The amount of lead in the umbilical cord blood was not shown to affect the gestational age and the anthropometry of the newborns. This is not in agreement with some previous studies in which lead levels had a negative correlation with gestation age and newborn anthropometry.[16,76,77,78] Rahman and Hakeem[79] on the other hand, reported no effect between the lead level and gestational age and newborn anthropometry similar to the finding in this study. In the current study, no associations were observed possibly because it was a cross sectional study carried out at the third trimester for most of the respondents. The small number of preterm babies and babies whose anthropometry were below the 3^rd^ centile may also be responsible as most of maternal obstetrics and socio-demographic factors also had no effect on the gestational age and anthropometry except lack of ANC that was significantly associated with OFC and maternal age of 35years or greater that significantly influence preterm birth. A prospective study with frequent sampling of mothers at different trimesters during pregnancy may be more appropriate to demonstrate the effect of lead on newborn anthropometry. This is because a single blood test may not be sufficient to establish the full nature of the developmental risk to the fetus or infant[3].

In conclusion, this study shows elevated lead level in both maternal and umbilical cord blood and demonstrable associated socio-demographic and obstetric factors. However, there were no associations between the lead levels and gestational age and newborn anthropometry. A longitudinal study on blood lead levels with follow up and repeat testing in both mothers and newborns will further help to advocate for screening and institution of appropriate interventions.

### Limitation

It was not possible to determine the duration of exposure of the neonates to high maternal lead levels as this was only measured at delivery; longer duration of exposure may have more significant effects on pregnancy outcome.

## Conclusion

Study highlighted the prevalence of elevated maternal and cord blood lead levels and adds to the existing body of evidence of high blood lead levels in Nigerians. There is therefore an urgent need to draft and enforce regulations on the manufacture, import, export, sale and distribution of lead containing products like paint and fuel in addition to educating the public on the hazardous effects of lead to control this problem.

## Supporting information

S1 File (DOCX)

S2 File (PDF)

S3 File (PDF)

S4 File (DOCX)
