# Supplementary material for: Determination of lead levels in maternal and umbilical cord blood at birth at the Lagos University Teaching Hospital, Lagos
Source: PLoS One. 2019 Feb 7;14(2):e0211535. doi: 10.1371/journal.pone.0211535 (PMC6366766; doi:10.1371/journal.pone.0211535)
Supplement: S1 File — (DOCX) [file pone.0211535.s001.docx]

**S1 Appendix. Study questionnaire**

RESEARCH QUESTIONNAIRE

MATERNAL DATA

1. INITIALS:
2. AGE:
3. STUDY I.D NUMBER:
4. HOSPITAL NUMBER:
5. OCCUPATION:
6. HUSBAND’S OCCUPATION:
7. HUSBAND’S AGE:
8. ADDRESS:
9. HOW LONG HAVE YOU BEEN LIVING AT THIS ADDRESS? :
10. RENOVATION (SCRAPPING OFF OLD PAINT AND REPAINTING) OF HOUSE IN THE LAST ONE YEAR: (a) yes (b) no
11. LAST MENSTRUAL PERIOD
12. GRAVIDITY
13. EDUCATIONAL LEVEL: (a) none (b) primary (c) secondary (d) tertiary
14. TRIBE:
15. REGISTERED FOR ANTE-NATAL IN A HEALTH FACILITY: (a) yes (b) no
16. ARE YOU ON CALCIUM SUPPLEMENTS?: (a) yes (b) no
17. DO YOU EAT NON FOOD SUBSTANCES SUCH AS CLAY, SAND, CHALK (PICA)?: (a) yes (b) no
18. IF YES TO NUMBER 17, WHAT DO YOU EAT?……………… AND HOW OFTEN DO YOU EAT IT?....................
19. HOW OFTEN DO YOU SERVE OR EAT FOOD SERVED IN CLAY POTS? (a) never (b)
20. DO YOU SMOKE CIGARETTES? : (a) yes (b) no
21. DOES ANY HOUSEHOLD MEMBER SMOKE CIGARETTES? : (a) yes (b) no
22. DO YOU DRINK ALCOHOL?: (a) yes (b) no
23. ARE YOU ON HERBAL MEDICATIONS/ ALTERNATIVE REMEDIES/ HERBAL TEAS?: (a) yes (b) no
24. AREA OF RESIDENCE:
25. IS YOUR HOUSE ON A MAJOR ROAD? :
26. WHAT IS THE SOURCE OF YOUR DRINKING WATER: (a)well (b) borehole (c) public supply (d) bottled water
27. DO YOU EAT CANNED FOODS: (a)yes (b) no
28. HAVE YOU BEEN DIAGNOSED WITH GESTATIONAL HYPERTENSION?: (a) yes (b) no
29. DO YOU USE ANY OF THESE EYE COSMETICS (KOHL, TIRO, AND SUMA)? (a) yes (b) no

TO BE COMPLETED BY RESEARCHER OR ASSISTANT

1. DURATION OF PREGNANCY IN WEEKS:
2. MATERNAL BLOOD LEAD LEVEL:

BABY’S DATA; (to be completed by the researcher or assistant)

1. SEX:
2. GESTATIONAL AGE:
3. BIRTH WEIGHT:
4. BIRTH LENGTH:
5. OCCIPITOFRONTAL CIRCUMFERENCE:
6. BABY’S CORD BLOOD LEAD LEVEL:
7. INTERVENTION:………………………………………………………………………………………………………………………………………………………………………………………………………………….
