# Supplementary material for: Determination of lead levels in maternal and umbilical cord blood at birth at the Lagos University Teaching Hospital, Lagos
Source: PLoS One. 2019 Feb 7;14(2):e0211535. doi: 10.1371/journal.pone.0211535 (PMC6366766; doi:10.1371/journal.pone.0211535)
Supplement: S3 File — (PDF) [file pone.0211535.s003.pdf]

[illegible]

HEALTH RESEARCH AND ETHICS COMMITTEE

PRIVATE MAIL BAG 12003, LAGOS, NIGERIA
e-mail address: luthethics@yahoo.com

Chairman
ASSOC. PROF. N. U. OKUBADEJO
MB. ChB, FMCP

Administrative Secretary
MR. D. J. AKPAN
B.Sc. BUS. ADMIN, MIHSAN

Chief Medical Director:
PROF. AKIN. OSIBOGUN
MBBS (Lagos), MPH (Columbia), FMCPH FWACP

Chairman, Medical Advisory Committee
DR. M. O. OGUNLEWE
BDS, FWACS.

LUTH HREC REGISTRATION NUMBER: NHREC: 19/12/2008a
Office Address: Room 107, 1st floor, LUTH Administrative Block
Telephone: 234-1-5850737, 5852187, 5852209, 5852158, 5852111

7th August, 2015

NOTICE OF EXTENSION OF RESEARCH APPROVAL DATE

PROJECT TITLE: "DETERMINATION OF LEAD LEVELS IN MATERNAL AND UMBILICAL CORD BLOOD AT BIRTH AT THE LAGOS UNIVERSITY TEACHING HOSPITAL, LAGOS".
HEALTH RESEARCH COMMITTEE ASSIGNED NO.: ADM/DCST/HREC/1910
NAME OF PRINCIPAL INVESTIGATOR: DR. LADELE JEJELOLA IBUKUNOLUWAPO
ADDRESS OF PRINCIPAL INVESTIGATOR: DEPT. OF PAEDIATRICS, LUTH.
DATE OF RECEIPT OF VALID APPLICATION: 23-06-14
DATE OF RE-APPLICATION FOR EXTENSION OF RESEARCH APPROVAL DATE: 06-08-15

This is to inform you that the research described in the submitted protocol, the consent forms, and all other related materials where relevant have been reviewed and given full approval by the Lagos University Teaching Hospital Health Research Ethics Committee (LUTHHREC).

This approval dates from 07-08-2015 to 07-08-2016. If there is delay in starting the research, please inform the HREC so that the dates of approval can be adjusted accordingly. Note that no participant accrual or activity related to this research may be conducted outside of this dates. All informed consent forms used in this study must carry the HREC assigned number and duration of HREC approval of the study. In multiyear research, endeavor to submit your annual report to the HREC early in order to obtain renewal of your approval and avoid disruption of your research.

The National code for Health Research Ethics requires you to comply with all institutional guidelines, rules and regulations and with the tenets of the code including ensuring that all adverse events are reported promptly to the HREC. No changes are permitted in the research without prior approval by the HREC except in circumstances outlined in the code. The HREC reserves the right to conduct compliance visits to your research site without previous notification.

HEALTH RESEARCH ... CHAIRMAN

PROF. N. U. OKUBADEJO
CHAIRMAN, LUTH HEALTH RESEARCH ETHICS COMMITTEE
