# Supplementary material for: Determination of lead levels in maternal and umbilical cord blood at birth at the Lagos University Teaching Hospital, Lagos
Source: PLoS One. 2019 Feb 7;14(2):e0211535. doi: 10.1371/journal.pone.0211535 (PMC6366766; doi:10.1371/journal.pone.0211535)
Supplement: S4 File — (DOCX) [file pone.0211535.s004.docx]

**APPENDIX I**

**PATIENT INFORMATION LEAFLET AND CONSENT CERTIFICATE.**

**STATEMENT OF INFORMED CONSENT**

TITLE: DETERMINATION OF MATERNAL AND UMBILICAL CORD BLOOD LEAD LEVELS AT BIRTH AT THE LAGOS UNIVERSITY TEACHING HOSPITAL, LAGOS.

INVESTIGATOR: DR LADELE JEJELOLA IBUKUNOLUWAPO.

I will like you and your baby to be a part of a research in our hospital. Details of the study will be explained to you before you make your decision. Participation is not compulsory and refusal to participate will not hinder or affect medical management of both you and your baby in this hospital. If you decide to participate, you can opt out of the study at any time if you change your mind.

INTRODUCTION:

Lead is poisonous to our bodies. Pregnant women and children are particularly more vulnerable to its effects. It can affect every organ in the body especially the developing brain in children. Mothers can transfer lead to their babies via the placenta during pregnancy. There is evidence that there is lead contamination in Lagos from sources such as water, air, paints, cosmetics, and other household products hence the possibility of elevated lead levels in your body. Mothers and babies found to have high levels would be managed appropriately.

PURPOSE OF STUDY:

The study is proposed to determine the blood lead levels of both mothers and newborns in Lagos State.

PROCEDURE:

Prior to delivery, 2.0mls of blood will be withdrawn from you to test your lead levels and another 2.0mls will be withdrawn from the umbilical cord at delivery for the same purpose. The lead test will be at no cost to you. A questionnaire designed to get information related to the study will be given to you to complete. If the blood lead levels are ≥5ug/dl, your baby will be followed up closely and will have a repeat blood lead test within a month. Values ≥25ug/dl will warrant a repeat test in 2 weeks and consultation with a specialist while values above 45ug/dl will require repeat within 24 hours and if still high appropriate treatment will be given. The results of the test will be communicated to you if you wish to have them.

POTENTIAL BENEFITS:

This will aid in prompt identification of mothers and babies at risk of lead poisoning or who are already poisoned and the initiation of appropriate management. It would also help to know the prevalence of elevated blood lead levels in this environment and to assess the effectiveness of the policies on prevention in the country.

RISKS:

The risk of this study to you is not beyond the slight pain you will feel while being pricked during blood sample collection.

RIGHTS:

You have a right to ask questions on any aspect of the study that is unclear to you before, during and after participation. You also have the right to withdraw from the study at any time without repercussions.

CONFIDENTIALITY:

I will keep the records of this study confidential. All information about you and your baby will be coded.

QUESTIONS- WHO TO CALL:

If you have any questions contact- Dr Ladele J.I, Department of Paediatrics, Lagos University Teaching Hospital, Lagos.

E –mail address: [jeje_ladele@yahoo.com](mailto:jeje_ladele@yahoo.com)

Phone number: 08036698026.

Or the Chairman,

LUTH Health Research and Ethics Committee

Room 107, Administrative building LUTH.

CONSENT:

By signing this form, you agree that you and baby should be part of this study.

CONSENT CERTIFICATE

I……………………………………………………………………………………. consent to participate in the above named research being conducted by Dr. Ladele Jejelola I from Department of Paediatrics, Lagos University Teaching Hospital, Idi-Araba, Lagos.

I have been given the following information:

- That this study is undertaken to determine the maternal and umbilical cord blood lead levels at the Lagos University Teaching Hospital.
- That blood lead testing will be done on both me and my baby.
- That I will answer some questions related to the research via questionnaires.
- That I will bear no cost of the test.
- That the risk of this study to me is not beyond pain I will experience during pricking.
- That this study will contribute to knowledge and add value to clinical practice.
- That the information obtained from me would be treated as confidential.
- That I have the right to ask for clarifications on any aspect of the study.
- That I have the right to withdraw from the study at any time without any consequence to me and my baby’s care in the hospital.

…………………………………………………………………

…………………………………………………………..

Signature / thumb print date
